# Genetic Analysis with the Immunochip Platform in Behçet Disease. Identification of Residues Associated in the HLA Class I Region and New Susceptibility Loci

**DOI:** 10.1371/journal.pone.0161305

**Published:** 2016-08-22

**Authors:** Lourdes Ortiz-Fernández, Francisco-David Carmona, Marco-Antonio Montes-Cano, José-Raúl García-Lozano, Marta Conde-Jaldón, Norberto Ortego-Centeno, María Jesús Castillo, Gerard Espinosa, Genaro Graña-Gil, Juan Sánchez-Bursón, María Rosa Juliá, Roser Solans, Ricardo Blanco, Ana-Celia Barnosi-Marín, Ricardo Gómez de la Torre, Patricia Fanlo, Mónica Rodríguez-Carballeira, Luis Rodríguez-Rodríguez, Teresa Camps, Santos Castañeda, Juan-Jose Alegre-Sancho, Javier Martín, María Francisca González-Escribano

**Affiliations:** 1 Department of Immunology, Hospital Universitario Virgen del Rocío (IBiS, CSIC, US), Sevilla, 41013, Spain; 2 Instituto de Parasitología y Biomedicina “López-Neyra”, CSIC, PTS Granada, Granada, 18016, Spain; 3 Department of Internal Medicine, Hospital Clínico San Cecilio, Granada, 18003, Spain; 4 Department of Internal Medicine, Hospital Universitario Virgen del Rocío, Sevilla, 41003, Spain; 5 Department Autoimmune Diseases, Hospital Universitari Clínic, Barcelona, 08036, Spain; 6 Department of Rheumatology, Complejo Hospitalario Universitario A Coruña, A Coruña, 15006, Spain; 7 Department of Rheumatology, Hospital Universitario de Valme, Sevilla, 41014, Spain; 8 Department of Immunology, Hospital Universitari Son Espases, Palma de Mallorca, 07120, Spain; 9 Department of Internal Medicine, Autoimmune Systemic Diseases Unit, Hospital Vall d’Hebron, Universidad Autonoma de Barcelona, Barcelona, 08035, Spain; 10 Department of Rheumatology, Hospital Universitario Marqués de Valdecilla, Santander, 39008, Spain; 11 Department of Internal Medicine, Complejo Hospitalario Torrecárdenas, Almería, 04009, Spain; 12 Department of Internal Medicine, Hospital Universitario Central de Asturias, Asturias, 33011, Spain; 13 Department of Internal Medicine, Hospital Virgen del Camino, Pamplona, 31008, Spain; 14 Deparment of Internal Medicine, Hospital Universitari Mútua Terrassa, Terrassa, 08221, Spain; 15 Department of Rheumatology, Hospital Clínico San Carlos, Madrid, 28040, Spain; 16 Department of Internal Medicine, Hospital Regional Universitario de Málaga, Málaga, 29010, Spain; 17 Department of Rheumatology, Hospital de la Princesa, IIS-Princesa, Madrid, 28006, Spain; 18 Department of Rheumatology, Hospital Universitario Doctor Peset, Valencia, 46017, Spain; Keio University, JAPAN

## Abstract

Behcet's disease (BD) is an immuno-mediated vasculitis in which knowledge of its etiology and genetic basis is limited. To improve the current knowledge, a genetic analysis performed with the Immunochip platform was carried out in a population from Spain. A discovery cohort comprising 278 BD cases and 1,517 unaffected controls were genotyped using the Immunochip platform. The validation step was performed on an independent replication cohort composed of 130 BD cases and 600 additional controls. The strongest association signals were observed in the HLA class I region, being HLA-B*51 the highest peak (overall P = 6.82E-32, OR = 3.82). A step-wise conditional logistic regression with classical alleles identified HLA-B*57 and HLA-A*03 as additional independent markers. The amino acid model that best explained the association, includes the position 97 of the HLA-B molecule and the position 66 of the HLA-A. Among the non-HLA loci, the most significant in the discovery analysis were: *IL23R* (rs10889664: P = 3.81E-12, OR = 2.00), the *JRKL/CNTN5* region (rs2848479: P = 5.00E-08, OR = 1.68) and *IL12A* (rs1874886: P = 6.67E-08, OR = 1.72), which were confirmed in the validation phase (*JRKL/CNTN5* rs2848479: P = 3.29E-10, OR = 1.66; *IL12A* rs1874886: P = 1.62E-08, OR = 1.61). Our results confirm HLA-B*51 as a primary-association marker in predisposition to BD and suggest additional independent signals within the class I region, specifically in the genes *HLA-A* and *HLA-B*. Regarding the non-HLA genes, in addition to *IL-23R*, previously reported in our population; *IL12A*, described in other populations, was found to be a BD susceptibility factor also in Spaniards; finally, a new associated *locus* was found in the *JRKL/CNTN5* region.

## Introduction

Behçet’s disease (BD) [MIM 109650] is a complex and immune-mediated systemic syndrome characterised by inflammatory lesions of various blood vessels throughout the body (though small vessels are most frequently involved), which lead to a wide range of clinical phenotypes such as recurrent oral and genital ulceration, ocular involvement (mainly uveitis) and skin lesions, amongst others. The aetiology of BD remains obscure, although some evidences suggest that certain infectious agents and environmental factors may trigger the disease in genetically predisposed individuals [1].

The particular geographical distribution of this type of vasculitis (along the ancient route known as ‘The Silk Road’, stretching from China to the Mediterranean area) [2], the reported familial aggregation and some specific associations of genes [3, 4], are data that together support a substantial genetic contribution to the pathogenesis of this disease. In this regard, as observed in most of the immune-mediated diseases, the region of the human Major Histocompatibility Complex (HLA) harbours the main genetic associations with BD. In fact, one of the class I molecule, specifically HLA-B51, has been repeatedly associated with disease predisposition in different ethnic groups, being considered the strongest genetic risk factor for this condition [4]. Others HLA class I molecules (including HLA-A, other HLA-B and HLA-C) have also been described as susceptibility markers for BD [5]. However, due to the strong linkage disequilibrium (LD) within the region as well as the limited statistical power of most studies, it is not clear whether they are independent signals or whether they are reflections of the primary association [5–7]. Besides, the identification of the most important amino acid positions that would be desirable for a better understanding of the functional implications of the HLA system in BD pathophysiology, have not yet been possible [6].

Outside the HLA region, there have been many studies of candidate genes in BD, but many of them have conflicting results and were not replicated in independent studies [8]. In addition, different genome-wide association studies (GWAS) have been performed in BD, including two in Turkish, one in Iranian, and three in Asian (Japanese, Chinese and Korean) populations [9–14]. The most significant non-HLA associations that are described in these studies, include *loci* that encode key immune components, such as *IL10*, the *IL23R-IL12RB2* region, *IL12A* and *STAT4*, thus confirming the crucial role that the immune system has in the development of this disease [5]. Despite what has been advanced, the rate of heritability that is explained by the currently established markers is still very low, therefore additional studies are required to unravel the genetic background underlying BD. One of the most successful platforms to explain part of the loss heritability when, as it is the case, the disease has a strong immunological component, is the Immunochip. This genetic platform is a custom high-density array that allows the analysis of 196,524 genetic variants across 186 susceptibility *loci* known for autoimmune and autoinflammatory disorders. Therefore, this is a high throughput genotyping platform with a candidate gene approach specifically designed for immunogenetic gene mapping [15]. The Immunochip has represented an important step forwards in the genetic study of other vasculitides, namely Takayasu arteritis [MIM 207600] and giant cell arteritis [MIM 187360] [16, 17], but also in the study of many other immune-mediated diseases like celiac disease [MIM 212750], rheumatoid arthritis [MIM 180300], psoriasis [MIM 177900], ankylosing spondylitis [MIM 106300], or systemic sclerosis [MIM 181750], amongst other [18–22]. Additionally, this array is particularly appropriate to extract precise information of the HLA system, due to the dense coverage of the fine-mapped *loci* within this region.

Considering the above and with the aim to contribute to improve the knowledge of the complex genetic bases of BD, a comprehensive analysis was performed in a case-control cohort of European ancestry from Spain using the Immunochip platform, with special emphasis on HLA region.

## Materials and Methods

### Study Population

**Fig 1:**
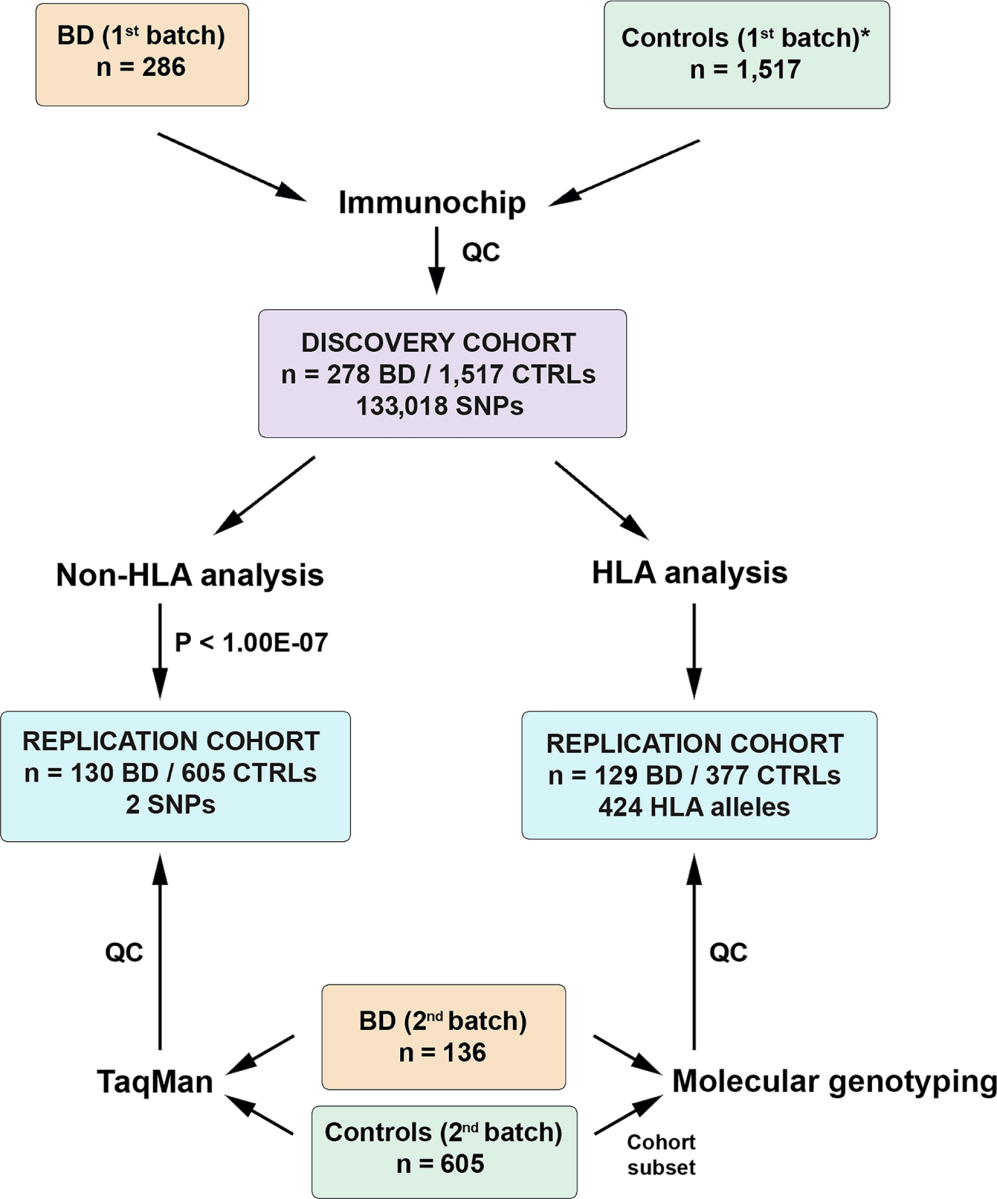
Workflow diagram summarising the methodological process of this study. *The control set was obtained from [17].

An overall population of 422 individuals diagnosed with BD (43.7% males) and 2,122 unrelated healthy controls (44.3% males) of Spanish European descent were included in this study. This cohort comprised an initial discovery group consisting of 286 newly genotyped BD patients and 1,517 healthy controls from a previously published study [17], as well as a replication group of an additional set of 136 BD cases and 605 controls (Fig 1). The BD cases were recruited from different Spanish hospitals across the country, and all of them fulfilled the 1990 International Study Group classification criteria for this disease [23]. Clinical features of the patient group were: 100% had oral ulcers, 75.3% skin lesions, 59.5% genital ulcers, 54% uveitis, 42% arthritis, 21% vascular, 18.2% neurological, 16.4% positive pathergy test and 15.5% gastrointestinal involvement. The study was approved by the ethical committees of all centres and hospitals involved: Hospital Universitario Virgen del Rocío, Sevilla, Spain; Instituto de Parasitología y Biomedicina “López-Neyra”, Granada, Spain;; Hospital Clínico San Cecilio, Granada, Spain; Hospital Universitari Clínic, Barcelona, Spain; Complejo Hospitalario Universitario A Coruña, A Coruña, Spain; Hospital Universitario de Valme; Sevilla, Spain; Hospital Universitari Son Espases, Palma de Mallorca, Spain; Hospital Vall d’Hebron, Barcelona, Spain; Hospital Universitario Marqués de Valdecilla, Santander, Spain; Complejo Hospitalario Torrecárdenas, Almería, Spain, Hospital Universitario Central de Asturias, Asturias, Spain; Hospital Virgen del Camino, Pamplona, Spain; Hospital Universitari Mútua Terrassa, Terrassa, Barcelona, Spain; Hospital Clínico San Carlos, Madrid, Spain; Hospital Regional Universitario de Málaga, Málaga Spain; Hospital de la Princesa, Madrid, Spain and Hospital Universitario Doctor Peset, Valencia, Spain. All participants signed a written informed consent prior to their enrolment in the study.

### DNA Extraction and Genotyping

Genomic DNA was extracted from 5 ml of peripheral blood collected in EDTA tubes using the ‘QIAmp DNA Mini Kit’ (Qiagen, Barcelona, Spain) following the manufacturer’s recommendations.

The BD cases from the discovery cohort were genotyped using the Immunochip array on the Illumina iScan system, accordingly with the Illumina protocol. Genotypes were called with the Genotyping Module (v.1.8.4) of the GenomeStudio Data Analysis software using the NCBI build 36 (hg18) mapping (Illumina manifest file Immuno_BeadChip_11419691_B.bpm). The replication phase of the suggestive non-HLA signals was performed using TaqMan® probes in a 7900HT Fast Real-Time PCR System (Applied Biosystems, Foster City, California, USA). To validate the results of the HLA analysis, the class I genes, *HLA-A*, *HLA-B* and *HLA-C*, were genotyped in the patient samples (n = 130) and in a subset of the control replication group (n = 377) using a standard low resolution (first set of digits) PCR-SSOP Luminex method (LABType SSO One Lambda Inc., Canoga Park, CA) in all the BD

### Quality Controls

Stringent quality control (QC) parameters were applied to all datasets prior to the analysis to control possible batch effects. The Immunochip raw data were filtered as follow: 1) The single-nucleotide polymorphisms (SNP) with a genotyping call rate lower than 95% and those that were not in Hardy-Weinberg equilibrium (HWE; P<0.001) were removed; 2) those samples with less than 90% of successfully called SNPs were discarded; 3) to prevent population stratification effects, we calculated the first five principal components (PC) from each individual with gcta64 and R-base under GNU Public license v.2. using ancestry informative markers of the Immunochip, and those samples deviating more than four standard deviations from the cluster centroids were considered outliers; and 4) duplicated samples or first-degree relatives were detected using the Genome function in PLINK v.1.07 [24] (Pi-HAT>0.4), and one sample per duplicate or relative pair were removed. After apply all these quality filters, information of 133,018 SNPs in 278 BD cases and 1,517 controls remained in the clean dataset of the discovery cohort. The Quantile–quantile plots were performed by housekeeping script and λ value for the discovery set was estimated at 1 (S1 Fig).

Regarding the validation stage, samples with genotyping call rates lower than 95% were filtered out from the replication cohorts, leading to a total number of cases/controls of 130/605 in this cohort. PC analyses could not be performed in this phase due to the lack of ancestry marker information. However, this case/control set was selected following an exhaustive clinical and demographic characterisation and its homogeneity has been confirmed in different studies published by our group [25, 26].

### Imputation of the HLA Region

Taking advantage of the high SNP coverage that the Immunochip has in the HLA region, the genotyping data from 20,000,000 to 40,000,000 base-pairs at chromosome 6 (which comprises the extended HLA region) obtained were used to impute SNPs, classical HLA alleles (both, with first and second sets of digits), and polymorphic amino acid positions with a previously validated method [27]. Briefly, the reference panel for the imputation was composed of 5,225 individuals of European origin with genotyping data for 8,961 genetic variants (common SNPs and INDEL polymorphisms) as well as types for HLA class I and II molecules at first and second set of digits resolution [28, 29]. The imputation process was performed with the SNP2HLA v1.0.3 package using the Beagle software [30, 31].

Imputed data was also subjected to tight quality filters. That is, only those variants successfully called in more than 95% of individuals that did not deviate from HWE (P<0.001) and samples with genotyping call rates greater than 95% were used for the analyses. The final set included a total of 7,261 SNPs, 424 classical HLA alleles (at first and second set of digits resolution) of class I (*HLA-A*, *HLA-B* and *HLA-C*) and class II (*HLA-DRB1*, *HLA-DQB1*, *HLA-DQA1*, *HLA-DPB1* and *HLA-DPA1*) genes, and 1,276 polymorphic amino acid variants (S1 Table).

In order to assess the accuracy of the imputation, we compared the imputed data for the HLA class I genes, *HLA-A*, *HLA-B* and *HLA-C*, of the BD cohort with those HLA genotypes obtained in the same individuals with the standard method described above. Two parameters were considered for this comparison 1) the accuracy, which is a measure of the reliability of the frequencies, and 2) a correlation coefficient (r) to establish reproducibility of the typing in each individual, as described elsewhere [27].

### Statistical Analyses

All statistical analyses were performed with PLINK and R-base. To test for association in the discovery phase, the variation of frequencies between cases and controls were compared means of logistic regression analyses using the five first PCs as covariates. Regarding the analysis of HLA, all the data: SNPs, classical HLA alleles (at first and second set of digits resolution) and all the possible combinations of amino acid residues per position were tested. The data from the replication cohort were also analysed by logistic regression but without adjusting for population stratification (see above). Then, both cohorts were meta-analysed using the inverse variance weighted method assuming a fixed effect model.

To identify independent effects in the HLA region, analysis dependency by logistic regression were conducted by conditioning on the top signals in a step-wise manner, as described [17]. In addition, the influence of each polymorphic amino acid position in disease susceptibility was also evaluated by performing likelihood ratio tests (omnibus) in R and using the first five PCs as covariates, as described [27].

Finally, we investigated the possible existence of non-additive effects of classical HLA alleles in our combined cohort, using the statistical framework described in [32]. In short, to avoid type I errors due to statistical power limitation and/or low imputation accuracy, we isolated in our dataset those classical allele-groups (first set of digits) of the HLA associated genes showing a frequency ≥ 0.5% and with at least ten homozygous subjects in the reference panel used for the imputation [28, 29]. Individuals carrying other allele-groups were excluded from the analysis. Then, we established a logistic regression model assuming disease risk on a log-additive scale (that is, an additive contribution from each classical HLA allele) and tested, by the means of a likelihood ratio test in R, the improvement in fit of a second model in which a dominance term for each HLA was included. In addition, to check whether the possible non-additive effects were a consequence of allelic interaction, the latter model was also compared against a third model containing an additive term for each allele and an interaction term between each pair of tested alleles by a likelihood ratio test.

The genome-wide level of significance (5.00E-08) was set as cut-off P-value to define significant associations outside the HLA region. For the analysis of classical HLA alleles and polymorphic amino acid variants of HLA molecules, the thresholds for statistical significance were established at 8.67E-03 and 3.91E-04, respectively. These P-values were calculated using the ‘Genetic Type I Error Calculator’ (GEG) software, which implements a Bonferroni-based validated method to control the genome-wide type I error rate at 0.05 [33].

Ribbon representations of the associated HLA molecules were constructed with the UCSF Chimera software [34].

## Results

**Fig 2:**
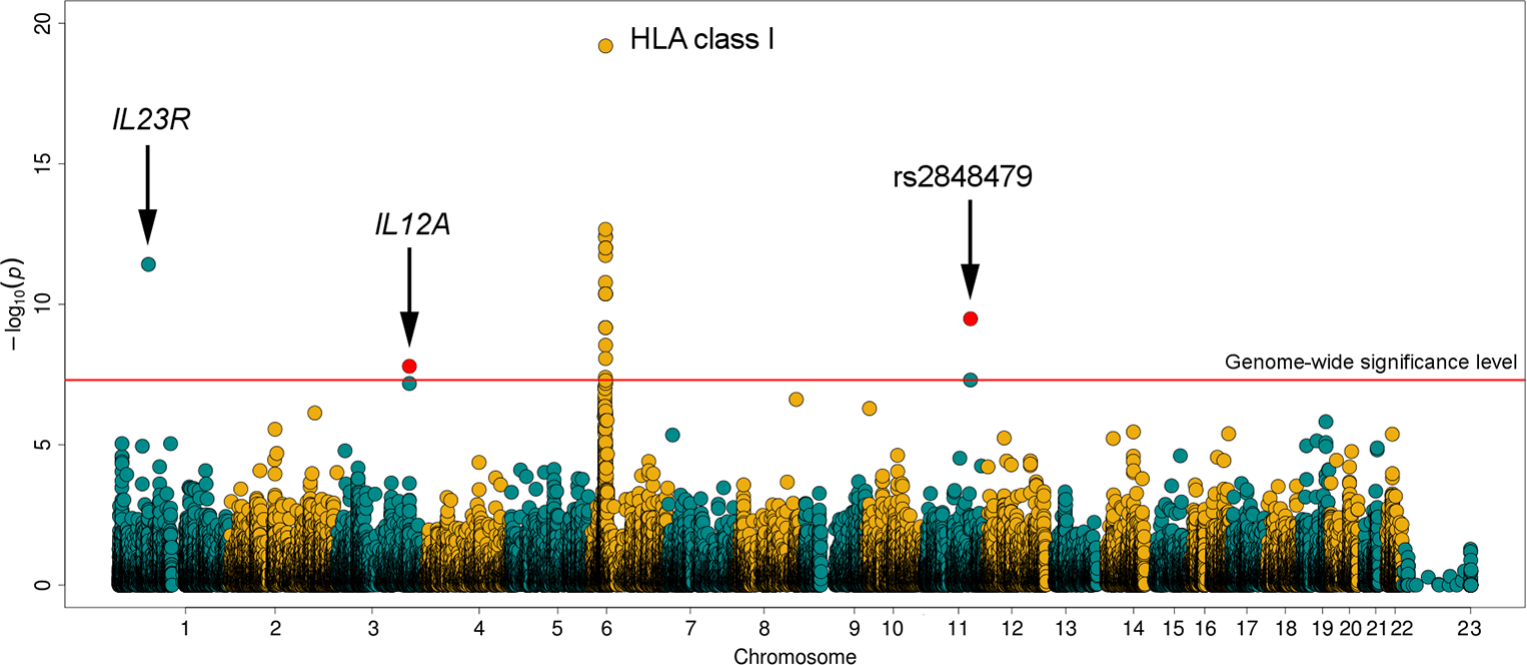
Manhattan plot representation of the Immunochip results. The–log10 of the combined logistic regression test P-values are plotted against its physical chromosomal position. The red line indicate the genome-wide level of significance (P<5x10E-08). The most relevant associations are highlighted. The red dots represent the overall results of *IL12A* and rs2848479 after the validation phase.

The strongest association signals with BD were located within the HLA region, although some suggestive signals were also detected in non-HLA *loci* (Fig 2).

### Interrogation of the HLA Region

To shed light into the HLA system contribution to disease risk, we decided to exhaustively analyse the HLA region by imputing: SNPs, classical alleles and polymorphic amino acid positions. A high overall accuracy of the imputation (>96%) was observed for the three HLA class I molecules (HLA-A, HLA-B and HLA-C) when comparing the best-guess genotypes (>0.9 probability) with those obtained for the same individuals using standard genotyping methods (S2 Table and S2 Fig). Additionally, the correlation coefficient (r) for the allele frequencies was also adequate, except for HLA-A*25 and HLA-A*26 that were inversely assigned in the imputation (*i*.*e*. HLA-A*25 individuals were called as HLA-A*26 and vice versa) (S2 Table).

To test for association within the HLA region, a logistic regression with the best-guess genotypes of all the imputed variants assuming an additive model was conducted, using the first five PCs as covariates to control for possible population stratification. Multiple association signals were detected within the class I region, with HLA-B*51 representing the highest peak (P = 5.56E-25, OR = 3.99, 95% CI = 3.07–5.19). However, no evidence of association was observed in any of the different HLA class II subregions (S3 Table and S3 Fig).

### Analysis of Classical HLA Alleles Assuming Additive Effects

The frequencies observed in the discovery cohort for all the imputed classical HLA (both at first and second set of digits) are displayed in S4 and S5 Tables. A step-wise conditional logistic regression analysis was then performed to identify those HLA alleles that independently influence the susceptibility to BD (Table 1). First, conditioning on HLA-B*51 the statistical significance of the class I signals was considerably reduced being HLA-B*57 the highest peak: (P = 1.02E-05, OR = 2.80, 95% CI = 1.77–4.43), which confirmed the major role that this HLA molecule seems to have in the immunopathological mechanisms leading to BD development. In a second step, we included both HLA-B*51 and HLA-B*57 as covariates in the analysis. When HLA-B*57 was added to the model, all the remaining classical alleles of HLA-B were non-significant, although some independent signals were still suggestive in HLA-A (top allele: HLA-A*03, P = 9.68E-03, OR = 0.61, 95% CI = 0.41–0.89). Finally, no additional independent associations were observed after conditioning on HLA-B*51, HLA-B*57 and HLA-A*03.

**Table 1:** Step-wise conditional analysis of imputed classical HLA alleles to detect independent associations with Behçet’s disease. OR, odds ratio; CI, confidence interval.

Covariates	Risk factor	P-value	OR (95% CI)
None	HLA-B*51	5.56E-25	3.99 (3.07–5.19)
HLA-B*51	HLA-B*57	1.02E-05	2.80 (1.77–4.43)
HLA-B*51+ HLA-B*57	HLA-A*03	9.68E-03	0.61 (0.41–0.89)

Next, to validate our findings, a replication cohort of 129 BD subjects and 377 unaffected controls was genotyped in HLA class I (HLA-A, HLA-B and HLA-C) using standard methods and, after that, data obtained from both cohorts were meta-analysed (S6 Table). The results of the replication step (HLA-B*51: P = 1.63E-08, OR = 3.42, 95% CI = 2.23–5.24; HLA-B*57: P = 9.99E-02, OR = 1.94, 95% CI = 0.88–4.26; HLA-A*03: P = 3.35E-02, OR = 0.54, 95% CI = 0.31–2.11) as well as those from the combined meta-analysis (HLA-B*51: P = 6.82E-32, OR = 3.82, 95% CI = 3.06–4.78; HLA-B*57: P = 2.43E-04, OR = 2.05, 95% CI = 1.40–3.02; HLA-A*03: P = 1.31E-04, OR = 0.55, 95% CI = 0.40–0.75) were consistent with the proposed model of, at least, three independent associated HLA class I molecules. The results of the step-wise conditional logistic regression analysis with the set of pooled data were the same that those obtained with the discovery cohort (data not shown).

### Analysis of Polymorphic Amino Acid Positions

**Fig 3:**
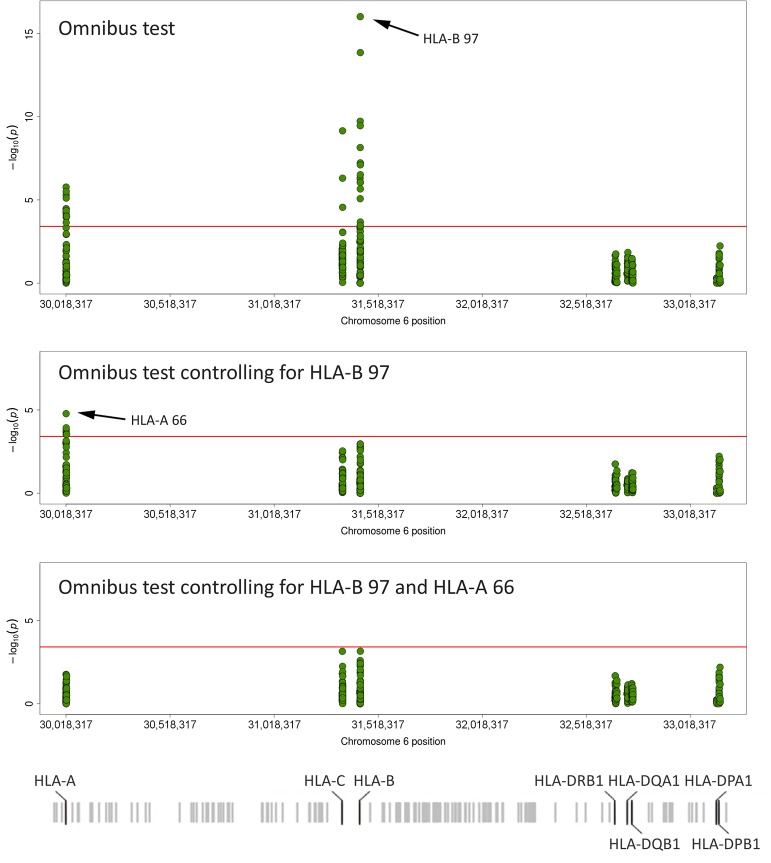
Manhattan plot representation of the Omnibus test on amino acid positions. A) Unconditioned test. B) Omnibus test conditioning on position 97 of HLA-B. C) Omnibus test conditioning on positions 97 and 66 of HLA-B and HLA-A, respectively. The–log10 of the combined logistic regression test P-values are plotted against the physical chromosomal positions of the centre of codon. The red line represents the threshold for statistical significance (P<3.91E-04).

In order to further investigate the HLA contribution to the pathogenesis of BD at the amino acid level, we carried out an omnibus test on polymorphic amino acid positions from the HLA molecules (Fig 3 and S7 Table). According to this analysis, the most relevant amino acid position for disease risk was the position 97 of HLA-B (likelihood P = 9.90E-17). Six possible residues were present at this position in the analysed cohort, with two of them conferring risk (Thr: P = 1.86E-16, OR = 2.68; and Val: P = 1.02E-03, OR = 2.09), other two conferring protection (Arg: P = 2.46E-08, OR = 0.58; and Ser: P = 1.49E-02, OR = 0.70, and the remaining two being neutral (Asn: P = 6.52E-01, OR = 0.87; and Trp: P = 8.21E-01, OR = 1.04) (Table 2). Most of the associated positions were explained by HLA-B 97 after performing the omnibus test using the amino acids at this position as conditioning factors. Only some positions of the HLA-A molecule remained significant (Fig 3 and S7 Table).

**Table 2:** The most relevant amino acid positions for the susceptibility to Behçet’s disease and those classical HLA variants (at resolution of the first set of digits) most common in our population (frequency >1%) containing the different residues at each position are shown.

**Molecule**	Position	Residue	Freq (BD)	Freq (CTRL)	*P*-value	OR (CI 95%)	Classical HLA alleles
HLA-B	97	Thr	0.29	0.14	1.86E-16	2.68 (2.12–3.39)	B*51
		Val	0.06	0.03	1.02E-03	2.09 (1.35–3.25)	B*57
		Arg	0.56	0.42	2.46E-08	0.58 (0.70–0.48)	B*13, B*15, B*18, B*35, B*38, B*39, B*40, B*44, B*45, B*49, B*50, B*53
		Ser	0.12	0.16	1.49E-02	0.70 (0.53–0.93)	B*07,B*08
		Asn	0.03	0.03	6.52E-01	0.87 (0.49–1.58)	B*27
		Trp	0.07	0.06	8.21E-01	1.04 (0.72–1.52)	B*14
HLA-A	66	Lys	0.49	0.37	1.80E-06	1.58 (1.31–1.90)	A*02, A*23, A*24
		Asn	0.51	0.63	1.80E-06	0.63 (0.77–0.53)	A*01, A*03, A*11, A*25, A*26, A*29, A*30, A*31, A*32, A*33

**Fig 4:**
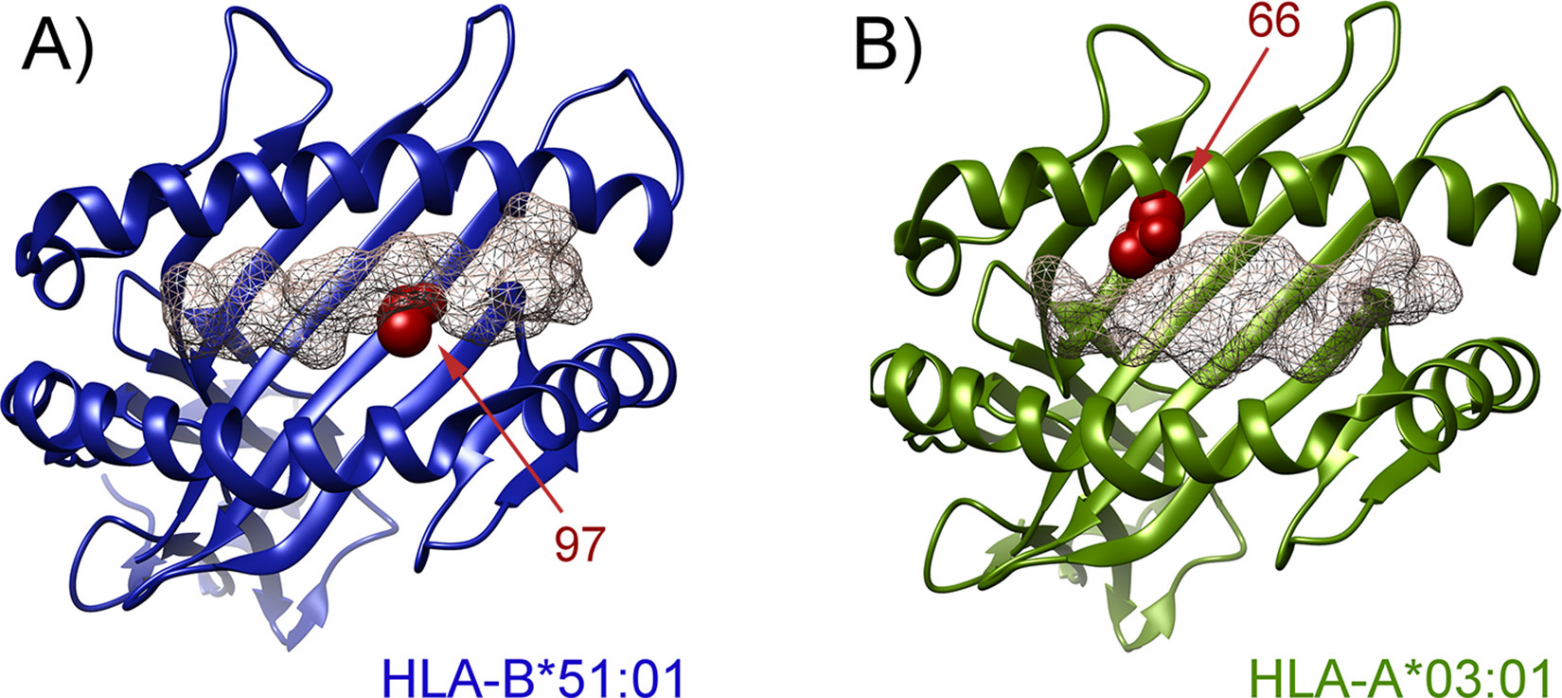
Ribbon representation of the HLA molecules HLA-B*5101 (A) and HLA-A*0301 (B). The independently associated amino acid positions with Behçet’s disease are highlighted in red. A mesh representation of the HIV immunodominant epitope KM1 and a proteolipid protein peptide are shown in HLA-B*5101 and HLA-A*0301, respectively, according to Protein Data Bank entries 1E27 [46] and 2XPG [37].

Regarding HLA-A, the position 66 showed the highest signal in the unconditioned omnibus test (likelihood P = 1.70E-06). Two amino acid variants can be present in this position, Lys (conferring risk, P = 1.80E-06, OR = 1.58) and Asn (conferring protection, P = 1.80E-06, OR = 0.63) (Table 2). As stated before, this association maintained the statistical significance after conditioning on HLA-B 97 (conditioned P = 1.67E-05). Moreover, when the dependency analysis was conducted considering both HLA-B 97 and HLA-A 66, none of the other associations were maintained (Fig 3 and S7 Table). Interestingly, these class I positions are located in the binding groove of the corresponding molecules (Fig 4), which adds a functional relevance to our data.

### Non-additive Contribution and Combined Effect of the HLA-A and B Molecules

We speculated whether possible non-additive effects of HLA variants could be influencing BD predisposition, as it has been proposed for other immune-mediated diseases [32]. Taking into consideration that all the observed HLA associations with BD were located in class I, specifically in the HLA-A and HLA-B molecules, we focused our analysis on two-digit classical variants of these two genes. Across the ten common tested alleles, only HLA-A*03 showed evidence of a non-additive effect (S8 Table), meaning that heterozygosity may exert a higher effect than that expected. We also investigated whether interactions between the HLA-A variants might explain this finding, however, no interactions between the analysed variants was detected (S9 Table**).** Finally, a possible combined effect of the HLA-A and B variants included in this part of the study was assessed, nevertheless, none interaction was detected (S10 Table).

### Association Test in the No-HLA Region

Outside the HLA region, only one SNP surpassed the genome-wide significance level in the analysis of the discovery cohort (rs10889664: P = 3.81E-12, OR = 2.00, 95% CI = 1.65–2.42). This is a genetic variant of the *IL23R* gene, which is a well established susceptibility marker for BD in different ethnic groups including the Spanish population [10, 11, 35].

However, many polymorphisms were observed within the grey zone showing suggestive P-values (<1.00E-05) (Fig 2 and S11 Table). Particularly, two of them were close to the statistical threshold, these are, rs2848479 in the *JRKL/CNTN5* region at chromosome 11 (P = 5.00E-08, OR = 1.65, 95% CI = 1.38–1.98) and the *IL12A* variant rs1874886 (P = 6.67E-08, OR = 1.67, 95% CI = 1.39–2.02). Consequently, we decided to genotype these two SNPs in a replication cohort composed of 130 BD cases and 605 healthy controls to confirm whether they represented real association signals in our population. Both SNPs were associated with BD predisposition at the nominal level in the replication set (*JRKL/CNTN5* rs2848479: P = 1.75E-03, OR = 1.63, 95% CI = 1.19–2.20; *IL12A* rs1874886: P = 3.97E-02, OR = 1.37, 95% CI = 1.02–1.85), and at the genome-wide level of significance in the meta-analysis of the discovery and replication cohorts (*JRKL/CNTN5* rs2848479: P = 3.29E-10, OR = 1.66, 95% CI = 1.42–1.94; *IL12A* rs1874886: P = 1.62E-08, OR = 1.61, 95% CI = 1.36–1.89) (Table 3).

**Table 3:** Novel genetic associations with Behçet’s disease at the genome-wide level of significance in the Spanish population. Results of the discovery, replication and combined analyses are shown. Chr, chromosome; Freq, allele frequency; OR, odds ratio for the minor allele; CI, confidence interval; BD, Behçet’s disease; CTRL, controls.

				DISCOVERY				REPLICATION				COMBINED			
				Freq BD	Freq CTRL		OR	Freq BD	Freq CTRL		OR	Freq BD	Freq CTRL		OR
Chr	Gene	Variant	Allele	(n = 278)	(n = 1,517)	P-value	(95% CI)	(n = 130)	(n = 605)	P-value	(95% CI)	(n = 408)	(n = 2,122)	P-value	(95% CI)
11	*JRKL / CNTN5*	rs2848479	A	0.529	0.404	5.00E-08	1.68	0.505	0.391	1.75E-03	1.63	0.523	0.401	3.29E-10	1.66
							(1.39–2.02)				(1.19–2.20)				(1.42–1.94)
3	*IL12A*	rs1874886	A	0.492	0.367	6.67E-08	1.72	0.447	0.378	3.97E-02	1.37	0.478	0.370	1.62E-08	1.61
							(1.41–2.09)				(1.02–1.85)				(1.36–1.89)

## Discussion

The present study provides firm evidence of an independent effect on BD predisposition for the HLA class I genes *HLA-A* and *HLA-B* in the Spanish population. Additionally, our results do not support the existence of independently associated signals within *HLA-C*, *MICA*, or any class II *locus*. In this regard, they are in agreement with those recently reported by Ombrello *et al*. [6] for the Turkish population, and contrary to the study on Turks and Italians by Hughes *et al* [7], as we did not find any genetic variant across the *HLA-B-MICA* region that could account for the robust HLA-B*51 association by itself (including the proposed SNP rs116799036).

Different circumstances make it difficult the attribution of disease association to a specific causal variant within the HLA system, including the broad LD throughout the region, the strong effect size of the reported associations with autoimmune processes, and the high relevance that the genes located within the region have in the immune response. To better understand the complex HLA association with BD, we have followed a comprehensive approach that combine the high-throughput genomics with the novel imputation methods to facilitate a deep interrogation of this genomic region [36]. In relation to the analysis of classical HLA alleles, besides the primary association of HLA-B*51, we identified HLA-B*57 as an independent risk factor for BD in Spaniards, consistent with the observations by Ombrello *et al*. [6] in Turks. However, we were not able to validate the independency of the associations that they reported for HLA-B*15, *27 and *49, which were not even associated in our study cohort. The discrepancy could be due to a possible limitation in the statistical power of our study to detect those signals and, therefore, a possible role for these alleles in the Spanish population may not be ruled out.

Regarding the HLA-A molecule, our analyses showed that HLA-A*03 is independently protective against BD in our dataset, similar to that observed in the Turkish population [6]. Interestingly, our data suggest that this allele could exert non-additive dominant effects that may explain a fraction of the phenotypic variance. We also detected a strong effect for HLA-A*02 as a risk variant, although the association was lost in the dependency analysis. Interestingly, the position 66 of the HLA-A molecule, one of the two positions comprising the amino acid model that better explained the HLA association with BD, differentiate both HLA-A molecules. The position 66 is a biallelic variant that may contain Asn or Lys. The former residue is present, among others, in HLA-A3 molecules, whereas the HLA-A2 molecules have the latter. This position is located in the binding groove of the molecule, being one of the most significant residues affecting the binding specificity of the B pocket of the HLA molecule [37]. In this context, recent evidences suggest that the position 66 of the HLA-A may influence the associated risk of autoimmune and autoinflammatory processes or the response to treatments [37, 38]. This motif was not included, however, in the model derived by Ombrello *et al*. to explain the association of the HLA class I alleles with BD in Turkey [6].

The analysis of polymorphic amino acid positions additionally confirmed the high relevance of the position 97 of the HLA-B molecule in BD. This position represented the strongest association signal in the omnibus test, as it was the case in the Turkish study [6]. It should be noted that the two most associated amino acids of this position (Thr and Val) characterise each of the two independent HLA-B molecules associated in our study (HLA-B51 and HLA-B57), thus supporting the reliability of our results. Additionally, the improvement of fit of the models including the top HLA-C positions were considerably reduced when the residues 97 of HLA-B were included as conditioning factors, consistent with the results observed in the step-wise conditional analysis of classical alleles. Hence, our results do not support the genetic association between HLA-Cw*1602 and BD pathogenesis identified by Hughes *et al*. [7].

On the other hand, although the position 97 explained all the observed signals within the HLA-B molecule, however, this may be taken cautiously, as this study could be underpowered to detect additional independent effects as reported by Ombrello et al. [6]. In addition, it is evident that each specific HLA-B molecule has a defined set of amino acids in its polymorphic positions, with many of them in LD thus increasing the difficulty to evaluate dependency at the amino acid level. It should be also noted that dependency does not exclude a possible influence. Therefore, only by complementing the knowledge gained by this type of approaches with those provided by functional studies, it would be possible to elucidate the precise etiopathogenic role of these molecules in disease, which would be essential for a personalised medicine [39]. In this sense, the second most associated position in our study was the position 80 of HLA-B. This position is included in the serologically defined epitope Bw4 (determined by the amino acid positions 79–83 of HLA-B), which is essential in the interaction with an inhibitory receptor of the NK cells (KIR3DL1) [40]. This interaction has an inhibitory effect on the cytotoxic capacity of NK cells [41]. Regarding this, it has been described that changes at specific positions of the HLA-B outside the Bw4 epitope, in particular position 97, affect the interaction of Bw4 with KIR3DL1 [40]. Altogether, the above suggests that both positions of the HLA-B molecule, 80 and 97, could be relevant in the susceptibility to disease.

With regards to the non-HLA region, no previous study has reported data using the Immunochip platform [5]. The peak association with BD outside the HLA region corresponded with a genetic polymorphism of *IL23R*, a crucial molecule in the immune response [42]. The involvement of this gene in this type of vasculitis is not novel, as it has been confirmed in different independent cohorts, including the Spanish population [10, 11, 35].

In relation to the *IL12A* association, this gene encodes a subunit of the heterodimeric cytokines IL-12 and IL-35 [43]. Previous studies have proposed *IL12A* as putative marker for BD development, including a GWAS in Turks and a meta-analysis of different populations [14, 44]. The SNP associated in the GWAS (rs17810546) was not included in the Immunochip, and it is not in LD with the associated variant in this study (rs1874886). In any case, although further studies are needed to fine-map this association, our data confirmed that *IL-12A* has a major role in BD also in Spaniards.

Finally, we have identified and validated in a replication cohort a novel association with BD at the genome-wide level of significance. The associated SNP (rs2848479) maps to an intergenic region between the human homolog of mouse jerky gene (*HHMJG*, *JRKL*) and the contactin 5 (*CTCN5*) gene. *JRKL* encodes a protein with an unknown function, whereas *CTCN5* encodes a member of the contactin family (belonging to the immunoglobulin superfamily) that mediates cell surface interactions during nervous system development [45]. Therefore, no clear functional implication can be inferred from our data regarding this association. It could be speculated that the causal variant was related to a trans-regulatory element. Nevertheless, considering that this is the first time that an association with BD has been reported within this region, replication studies in other populations as well as functional studies are required to confirm this finding.

In conclusion, our study supports the existence of independent HLA associations with BD only within the class I genes *HLA-A* and *HLA-B*. Specifically, the classical variants HLA-B*51, HLA-B*57, and HLA-A*03 explained most of the genetic influence of the HLA system in the disease. We built a model of two amino acid positions with a putative functional effect, 97 in HLA-B and 66 in HLA-A, which is consistent with these associations. Moreover, this study confirms *IL23R* and *IL12A* as major contributors to disease susceptibility and proposed a novel genetic marker for BD, located in the *JRKL/CTCN5* region, at the genome-wide level of significance.

## Web Resources

The URLs for data presented herein are as follows:

1000 Genomes, http://www.1000genomes.org/

BEAGLE, http://faculty.washington.edu/browning/beagle/beagle.html

GEC, http://statgenpro.psychiatry.hku.hk/gec/

HLA Nomenclature, http://hla.alleles.org/

IMPUTE2, http://mathgen.stats.ox.ac.uk/impute/impute_v2.html

NCBI, http://www.ncbi.nlm.nih.gov/

Online Mendelian Inheritance in Man (OMIM), http://omim.org/

PLINK, http://pngu.mgh.harvard.edu/~purcell/plink/

RCSB Protein Data Bank, http://www.rcsb.org/pdb/home/home.do

SNP2HLA, https://www.broadinstitute.org/mpg/snp2hla/

UCSF Chimera, http://www.cgl.ucsf.edu/chimera/

## Supporting Information

S1 Fig Quantile–quantile plots of results of the association test for all SNPs after application of the quality filters (A) and of results of the association test excludingt the HLA region markers (B).
(TIF)

S2 Fig Imputation accuracy in this study of classical HLA-A, HLA-B and HLA-C alleles. (TIF)

S3 Fig Manhattan plot representation of the logistic regression of the imputed HLA region. The −log_10_ of the logistic regression test *P*-values are plotted against its physical chromosomal position. A red/blue colour gradient was used to represent the effect size of each analysed variant (red for risk and blue for protection). The diamond size depends on the linkage disequilibrium (r^2^) with HLA-B*51.
(TIF)

S1 Table HLA variations analysed in this study after imputation. (XLS)

S2 Table Imputation accuracy and correlation coeficient of the HLA class I allele-groups (first set of digits). The allelic accuracy represents the aggregate difference between the actual number and the imputed number of alleles observed. The r, Pearson product-moment correlation coefficient between dose of allele genotyped and imputed allele in each individual.
(XLS)

S3 Table Additive model association testing all the HLA variants analysed in this study. The marker nomenclature is as follow: 1) Classical HLA alleles: HLA_[gene]_[allele]; 2) HLA amino acids: AA_[gene]_[amino acid position within the molecule]_[genetic position of the center of codon]_[allele]; 3) HLA intragenic SNPs: SNP_[gene]_[genetic position]_[allele]; 4) Insertions/deletions: [variant]_[gene]_[genetic position]_[insertion/x = deletion]. For binary encodings, P = present, A = absent. In the case of amino acids, when more than one code is shown, this means the presence of any of them.
(XLS)

S4 Table Additive model association testing of classical HLA variants (first set of digits), HLA-A*25 and HLA-A*26 were not included in this analysis because of their low correlation coefficient (r), (XLS)

S5 Table Additive model association testing of classical HLA alleles at the second group of digits resolution, HLA-A*25 and HLA-A*26 alleles were not included in this analysis because of their low correlation coefficient (r), Only those HLA alleles found are displayed. (XLS)

S6 Table Validation study of classical HLA-A and HLA-B alleles at the first group of -digits resolution in an independent replication cohort. (XLS)

S7 Table Omnibus test of amino acid positions of the HLA molecules. Unconditioned and conditioned P-values on the top signals are shown. (XLS)

S8 Table Additive and non-additive effect sizes in the susceptibility to Behçet’s disease of common HLA-A and HLA-B alleles. (XLS)

S9 Table Analysis of interaction effects among HLA-A common alleles. (XLS)

S10 Table Analysis of combined effects among HLA-A and HLA-B common alleles. (XLS)

S11 Table Suggestive non-HLA signals (P<1,00E-4) with Behçet’s disease in the analysis of the discovery cohort, Variants selected for replication are highlited in bold. (XLS)
