# Supplementary figures and images for: Genetic Analysis with the Immunochip Platform in Behçet Disease. Identification of Residues Associated in the HLA Class I Region and New Susceptibility Loci
Source: PLoS One. 2016 Aug 22;11(8):e0161305. doi: 10.1371/journal.pone.0161305 (PMC4993481; doi:10.1371/journal.pone.0161305)

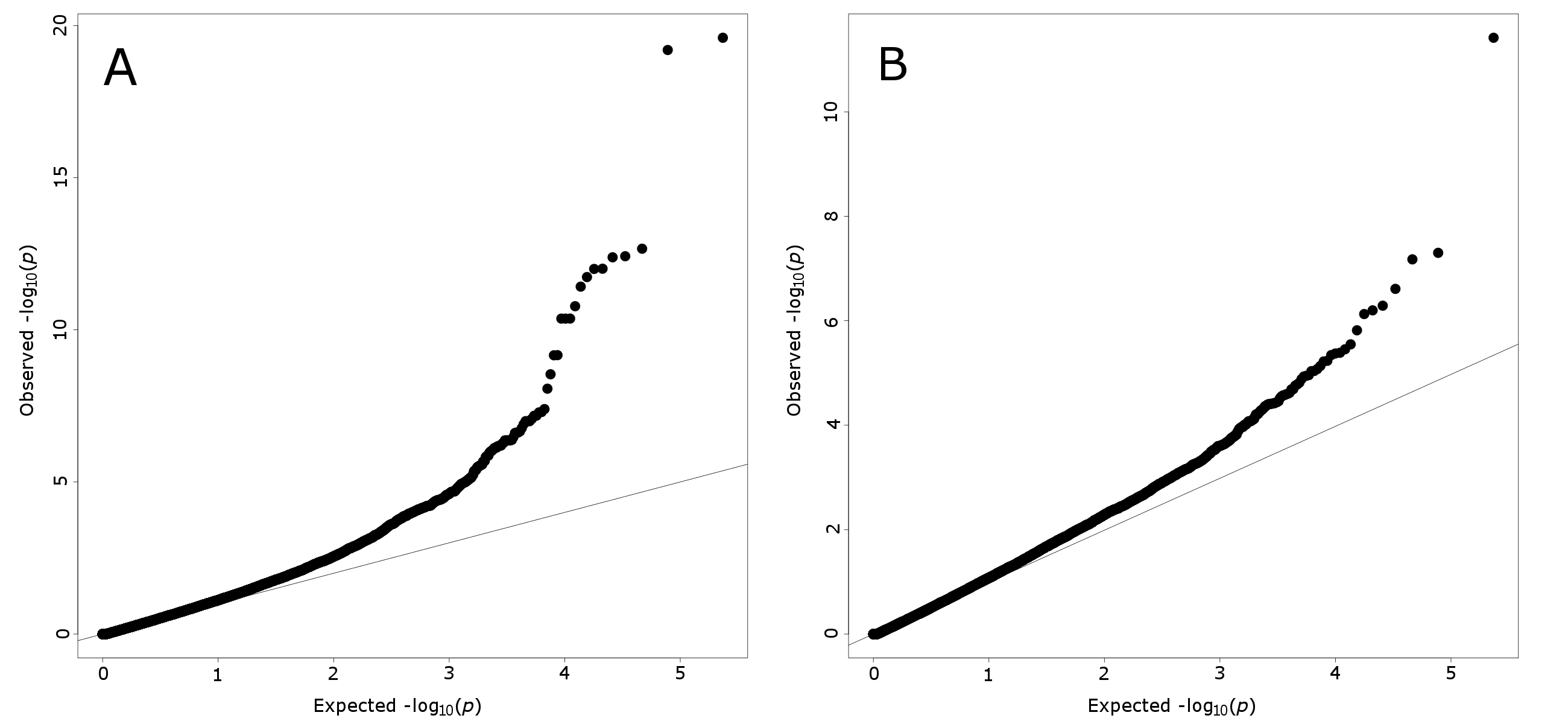

Supplement: S1 Fig — Quantile–quantile plots of results of the association test for all SNPs after application of the quality filters (A) and of results of the association test excludingt the HLA region markers (B). (TIF) [file pone.0161305.s001.tif]

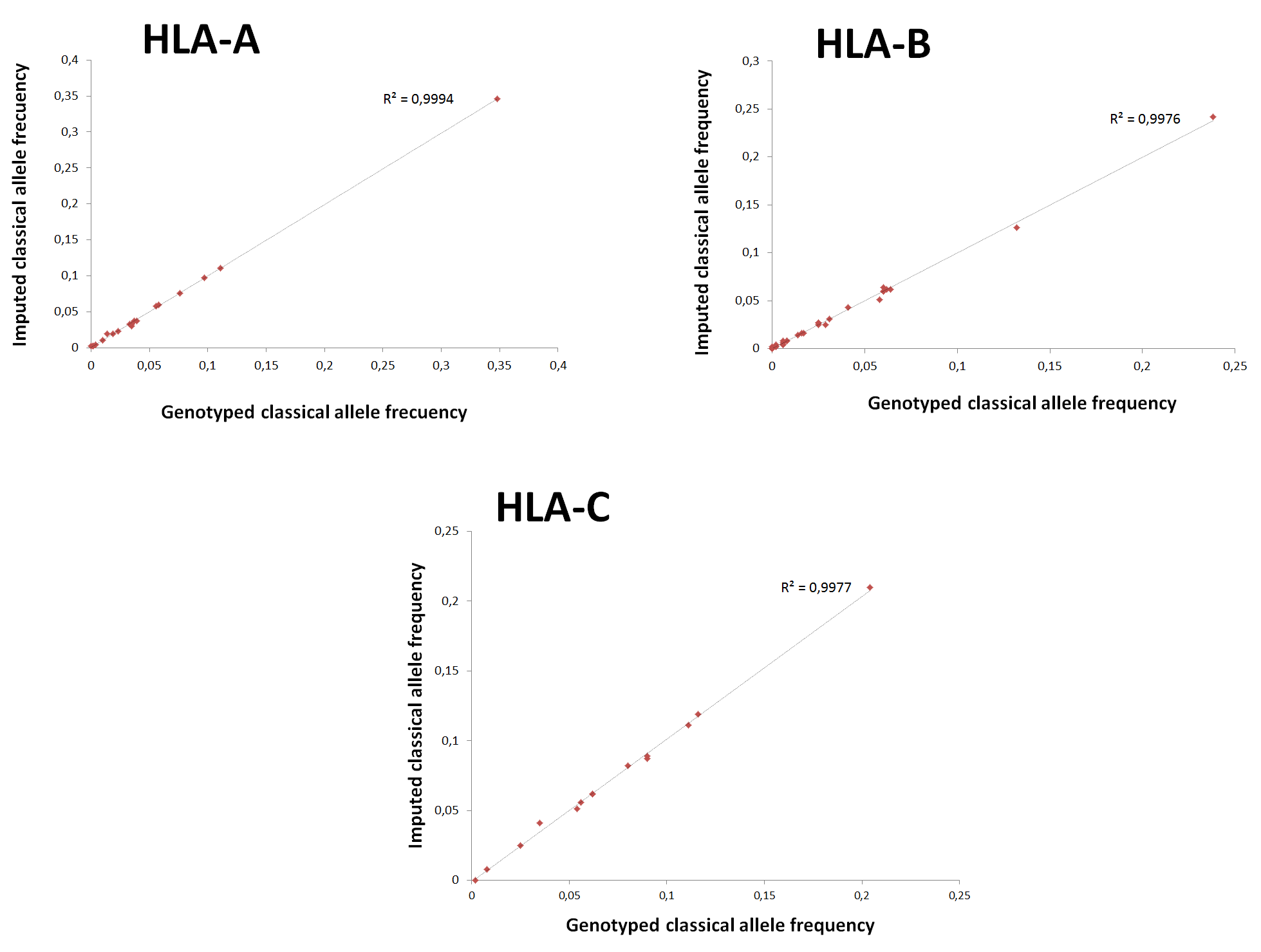

Supplement: S2 Fig — (TIF) [file pone.0161305.s002.tif]

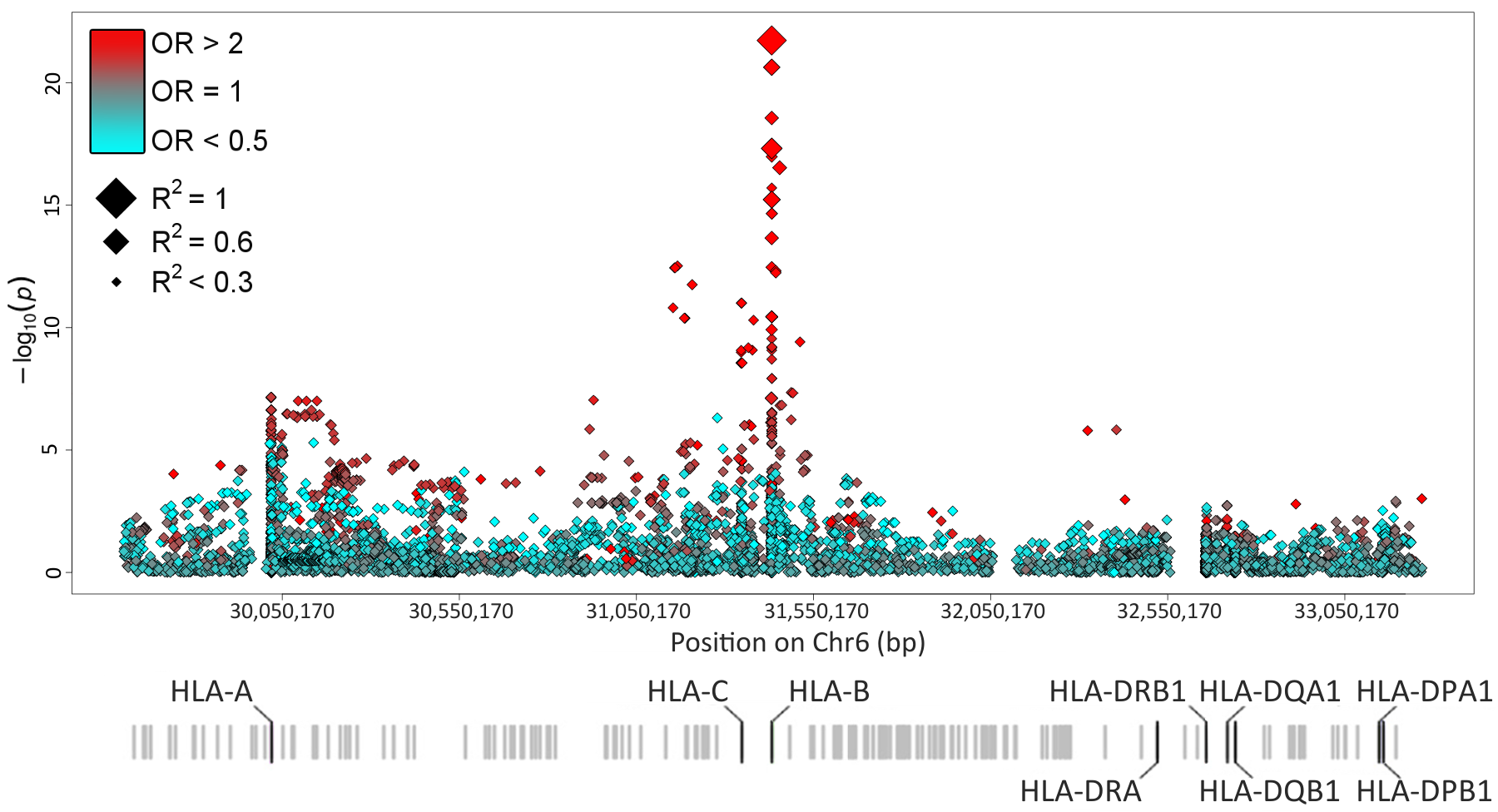

Supplement: S3 Fig — The −log10 of the logistic regression test P-values are plotted against its physical chromosomal position. A red/blue colour gradient was used to represent the effect size of each analysed variant (red for risk and blue for protection). The diamond size depends on the linkage disequilibrium (r2) with HLA-B*51. (TIF) [file pone.0161305.s003.tif]
